# Development and Validation of the Haze Risk Perception Scale and Influencing Factor Scale—A Study Based on College Students in Beijing

**DOI:** 10.3390/ijerph19084510

**Published:** 2022-04-08

**Authors:** Yongbao Zhang, Jianwu Chen, Xingfei Wei, Xiang Wu

**Affiliations:** 1 School of Management, Lanzhou University, Lanzhou 730000, China; zhangyb2021@lzu.edu.cn (Y.Z.); weixf2019@lzu.edu.cn (X.W.); 2 Institute of Occupational Health, Chinese Academy of Safety Science and Technology, Beijing 100012, China; 3 School of Engineering and Technology, China University of Geosciences (Beijing), Beijing 100083, China; wuxiang@cugb.edu.cn

**Keywords:** haze, risk perception, influencing factors, scale, college students in Beijing

## Abstract

Although Beijing’s air quality has improved, there is still a long way to go for haze governance. In order to understand haze risk perception and related influencing factors among college students in Beijing, we developed and verified two scales, with college students as the survey object, and analyzed the theoretical framework and realistic level of haze risk perception and influencing factors through empirical research. We showed that the reliability and validity of the two scales are excellent, and they can be used as a powerful tool to measure college students’ perception of haze. The haze risk perception scale (HRPS) is divided into four dimensions. The degrees of perception ranked from high to low are: direct consequences perception, indirect consequences perception, risk responsibility perception and risk source perception. The haze risk perception influencing factor scale (HRPIFS) is divided into three dimensions. The degrees of influence ranked from high to low are: personal emotion, media communication and government policy; the three influencing factors all have a significant positive correlation to overall haze risk perception, but personal emotions and media communication are only significantly related to the three dimensions of direct consequence perception, indirect consequence perception and risk source perception. Government policy is only significantly related to the three dimensions of direct consequence perception, indirect consequence perception and risk liability perception. This paper proves the important role of media in haze risk perception and puts forward some policy suggestions to guide the public to form a rational risk perception. These findings can help improve theoretical and practical research related to haze risk.

## 1. Introduction

In February 2015, the news investigation “Under the Dome Documentary” about haze aroused strong public attention from the Chinese people [1,2]. In this new stage of development, controlling air pollution has become the top priority of China’s green development concept [3]. Judging from the global real-time air quality index (https://aqicn.org/map/china/cn/#) accessed on 1 November 2020, Beijing, as the capital of China and an international metropolis, still has a long way to go to control haze [4]. Successful environmental risk management is inseparable from public understanding and the efforts of the whole society, which requires the government to fully understand public perception and the factors that may affect this perception when formulating haze policies [5]. As an active element in society, college students have a higher level of education and are at a critical stage in forming their perception of the external environment. College students will participate in all walks of life in the future and thus represent an important force in social governance. Scientific evaluation of college students’ haze risk perception and influencing factors can not only reflect the attitudes and emotions of college students towards haze, but also serve as an important reference for evaluating the effectiveness of haze governance. Therefore, it is worthy of in-depth study.

Risk perception is a process in which people judge the degree of danger of things based on their subjective feelings and then make relevant decisions [6]. Measuring public risk perception status through psychological measurement scales is an important basis for formulating and implementing reasonable risk management strategies [7]. Risk perception is a multi-dimensional concept, and in real life the public often shows two extremes: either excessive panic or indifference to haze risk [8]. This fully shows that there is a bias in haze risk perception among different groups, and this bias is affected by factors such as psychological characteristics, cultural background and social characteristics [9]. Therefore, it is of great theoretical and practical significance to develop a set of scientific measurement tools to qualitatively construct the structural elements of haze risk perception and its influencing factors, and to quantitatively evaluate the relationship between perception and the actual level of haze. However, previous studies on haze risk perception generally take the public as the research object and are not very targeted [10]. Therefore, research on haze risk perception not only needs to combine the haze risk situation and the characteristics of the subject group, but also needs to systematically build an evaluation framework of risk perception and related influencing factors to effectively reflect the characteristics and current situation of haze risk.

Based on this, this study combines risk perception theory with haze risk in the setting of China, and uses college students in Beijing as the survey object to develop and verify the haze-risk-perception scale (HRPS) and the haze-risk-perception influencing-factors scale (HRPIFS) for college students. In addition, the scales are used for empirical research to explore the actual level of college students’ haze risk perception and to analyze the impact of influencing factors to haze risk perception. Not only can this study guide college students to form a rational risk perception of haze, but it can also provide a theoretical basis for policy enlightenment for the government to improve the decision-making on haze risk governance.

## 2. Theories and Methods

### 2.1. Literature Review

In the research of risk perception, the classification and evaluation of risk perception factors always occupies an important position. Risk perception can be explained by a psychometric paradigm, in which the perception of risk is mapped to a factor space defined by multiple dimensions [11,12]. The classic risk perception theory proposed by Slovic P presents that risk perception has two dimensions: fearful risk and unknown risk. The former focuses on explaining the severity of the risk, and the latter focuses on mastery of the basic knowledge of risk [6]. With the continuous development of risk research and its application in different fields, risk perception has shown more differentiated dimensions. Carolyn Kousky followed housing prices in areas that had suffered severe flooding over 27 years and concluded that risk events are a key factor in public risk perception [13]. Studies have also shown that extreme weather events are an effective way for college students to perceive the risks of climate change [14]. Another important dimension of risk perception is the degree of understanding of risk knowledge, of which the perception of risk source is the most representative [10,15,16]. In addition to classic fear and unknown dimensions, the risk perception scale constructed by Paolo Gardoni also includes the dimension of risk source perception [17]. Combining the classic risk perception dimensions and the characteristics of haze risk, the perception of risk consequences seems to be an important factor that cannot be ignored [16,18]. Rickard and Laura N linked risk perception with attribution theory and proposed that public perception of risk is closely related to attribution of risk responsibility [12]. In the field of environmental risk, there have been studies that regard the perception of risk responsibility as an important dimension of risk perception [19,20].

The public’s risk perception based on the external risk environment is influenced and determined by various factors [21]. Previous research on the COVID-19 pandemic has shown that personal emotions are an important factor in risk perception, and negative emotions often mean more serious risk perception [22]. Terpstra Teun believes that in flood disasters, personal emotions can also predict and affect the level of risk perception, and, based on the research results, he proposed cognitive and emotional mechanisms that affect the public’s disaster preparedness intentions [23]. With the continuous development of informatization, social media has become an important channel for disseminating risk information. Studies have shown that personal health concepts and behaviors in risk situations are highly influenced by media information [24]. Taking the haze issue as an example, receiving information from social media will significantly increase peoples’ willingness to wear anti-haze masks [25]. In addition, related studies have shown that the intervention of mass media and social networking sites not only affects public perception of haze risk, but also promotes the formulation and implementation of more effective environmental protection policies [26,27]. The public often judges the risks posed by potentially hazardous facilities based on the government, industry experts and online media [28]. Studies have shown that the more the public distrusts the government, risk managers and risk facility operators, the higher their perceived risks, and the stronger their will to fight things. Therefore, many scholars regard social trust as one of the key indicators of public risk perception [29]. Zhang Yongbao’s research shows that public risk perception will change significantly (positively or negatively) with major government decisions [22]. Wang Lingling compared the haze risk perception of residents of China in areas with or without air improvement policies, and proved that government policy affects the public’s haze risk perception [30]. Through literature review, we found that—whether it is a psychological–cognitive paradigm or a social–cultural paradigm—scholars generally believe that personal emotion [31,32], media communication [33], social trust [22] and government policy [34] significantly affect public risk perception.

Early research on haze mainly focused on natural science aspects, such as the pollution formation mechanisms [35], human health loss [36,37] and environmental hazard assessment [38]. With the expansion of the impact of haze on society and the enhancement of people’s concept of green development, the social science attribute of haze research has gradually increased. Especially in recent years, haze risk has become one of the research hotspots in the fields of policy, psychology and management [30,39]. However, existing research on the risk perception of haze is not comprehensive enough. From the perspective of fields of research, macro research accounts for a large proportion, and research on individual cities and special groups is less prominent. Research content often focuses on some dimensions of haze risk perception, but discussion of factors influencing risk perception is relatively scarce, and research integrity and systems are insufficient. In addition, many research results on risk perception come from a single round of surveys, and data reliability is not high [14]. Taking college students as the survey object to explore their risk perception and influencing factors has been studied in other fields, such as the COVID-19 pandemic [40], but there is still a lack of research on the study of college students in a certain area for haze risks.

College students are at the critical stage of understanding society. They have a high degree of education and a strong willingness to participate in society, especially on the internet. As the backbone of the future society, a scientific and accurate evaluation of college students’ haze risk perception and influencing factors would not only reflect the social status quo, but also have guiding significance for improving air protection policies. Therefore, we developed and validated scales to clarify the constituent elements and influencing factors of haze risk perception among college students in Beijing. Then, through empirical research, we measured the realistic level of haze risk perception and provided decision-making references for formulating targeted haze governance strategies.

### 2.2. Theoretical Construction

#### 2.2.1. Haze Risk Perception

**Figure 1:**
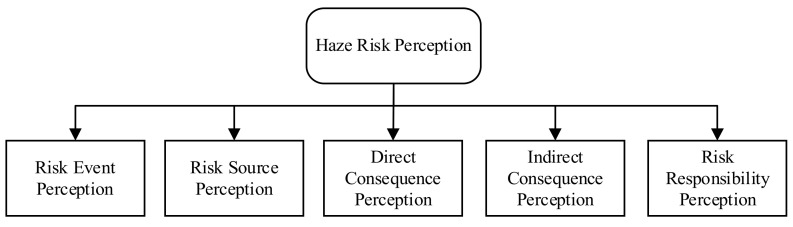
The structure of haze risk perception.

Through literature review and expert consultation, we believe that four dimensions—risk event perception, risk source perception, risk consequence perception and risk responsibility perception—can be used to measure haze risk perception. On this basis, we subdivide risk consequence perception into direct consequence perception and indirect consequence perception, and formulate five dimensions, as shown in the Figure 1.

Haze risk events measures college students’ perception of some representative events caused by haze; haze risk source perception measures college students’ mastery of the basic knowledge of haze, mainly through understanding the causes of haze, to reflect the perception level of haze risk sources; perception of direct and indirect consequences of haze mainly discusses the adverse effects of haze, including two parts: impact on human health and impact on society; haze risk responsibility perception mainly discusses the responsibility of enterprises and governments in the process of haze generation and governance.

#### 2.2.2. Influencing Factors of Haze Risk Perception

**Figure 2:**
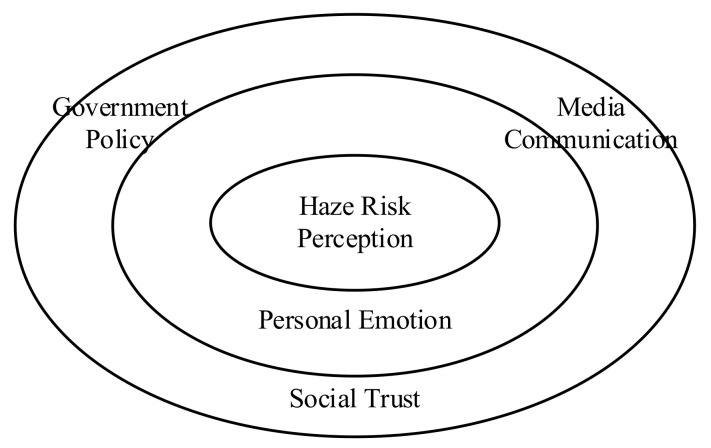
The relationship between haze risk perception and influencing factors.

The protective action decision model (PADM) considers that people’s risk perception is derived from external environmental information related to the harm they have suffered and based on previous personal experience [41]. Therefore, people’s risk perception is influenced by their self-identity as well as their communication and trust with the external environment [42]. Through the literature review above, we believe that personal emotion, media communication, social trust and government policy can be used as influencing factors of haze risk perception. According to PADM, we tried to construct the relationship between haze risk perception and influencing factors, as shown in Figure 2.

The center in the figure is haze risk perception, the middle layer is personal emotions, and the outer layer is media communication, social trust and government policy. We believe that haze risk perception is directly affected by personal emotion, and then indirectly affected by external media communication, social trust and government policy.

### 2.3. Research Tools and Methods

#### 2.3.1. Scale Design

Based on literature review and theoretical construction, we took college students in Beijing as the survey object and compiled the HRPS and the HRPIFS for college students. The questionnaire consists of three parts. The first part explains the purpose of collecting data in this questionnaire. The second part is demographic information, which mainly records the gender, age and educational level of the college students. The third part measures the college students’ risk perception of haze and the influencing factors of risk perception. The initial HRPS has a total of 5 dimensions and 15 items, and the initial HRPIFS has a total of 4 dimensions and 15 items. The content of the scale is based on suggestions from doctoral students in psychology, management and environmental science, but at this time the content and structure of the scale have not been finalized. The final scale can be determined only after subsequent systematic investigation and data analysis.

The scale uses the Likert five-point scoring method, with five options “strongly disagree,” “disagree,” “unclear,” “agree” and “strongly agree,” corresponding to 1–5 points, respectively. The higher the score, the higher the level of risk perception of college students or the stronger the importance of influencing factors.

The questionnaire survey is generally divided into two stages: development and validation. In the development stage, two questionnaires were used to independently investigate the haze risk perception and the influencing factors of risk perception. In the validation stage, the two research topics were investigated in one questionnaire. All questionnaires were distributed online. Participants in the development stage were students from The China University of Geosciences (Beijing), and participants in the validation stage were college students in Beijing.

#### 2.3.2. Data Analysis Method

The scales in the development and validation stages were analyzed by SPSS 22.0 and Amos 26.0 software developed by IBM, New York, NY, USA, respectively. The analysis process of the initial scales mainly included item analysis, exploratory factor analysis (EFA) and reliability analysis. Item analysis used the extreme group *t*-test method to analyze the discrimination of each item [43]. Specifically, we summed the scores of all items and then sorted them according to the total score. We defined 27% of the total score from this item as the cutoff values for the high and low groups. If there was no significant difference between the high and low group on each item, the item was deleted. EFA is usually tested by principal component analysis (PCA), and orthogonal rotation maximizes variances (varimax) [44]. Through KMO coefficient and Bartlett test, factor loading, scree plot, communality, Cronbach’s Alpha and other indicators, the substandard dimensions and items were deleted. The KMO coefficient and Bartlett test were used to analyze the validity of the scale data: the larger the KMO coefficient, the more suitable for factor analysis. Cronbach’s Alpha, also known as the internal consistency coefficient, is an important indicator of reliability. A larger Alpha means better reliability of the scale [45].

The analysis in the validation stage mainly included confirmatory factor analysis (CFA), reliability analysis and validity analysis. CFA uses the maximum likelihood method to test the structure of scales [46]. If the factor loading, commonality, fitting results of CFA, KMO coefficient and Bartlett test, Cronbach’s Alpha, common method variance (CMV), average variance extracted (AVE) and combined reliability (CR) are not up to standard in the validation stage, the structure and content of the scales need to be improved. The reliability analysis method is the same as EFA. Comprehensive validity tests usually include construct validity, content validity, convergent validity and discriminant validity [47]. KMO and Bartlett’s test can reflect the construct validity. Content validity requires careful consideration in the early stages of scale design. Strictly speaking, convergent validity and discriminant validity are subtypes of construct validity, but with different indicators. Convergent validity uses AVE and CR values to test the ability of items to measure the same factor, and discriminant validity uses the heterotrait–monotrait ratio (HTMT) to test the difference between items of different factors.

In the empirical research, we successively used proportional analysis, mean analysis, bivariate correlation analysis and variance analysis. Through large sample data, it is possible to understand the realistic level and internal connections of college students’ risk perception of haze and its influencing factors.

## 3. Results

### 3.1. Initial Test Results of the Scales

#### 3.1.1. Development of the Initial HRPS and HRPIFS

Since the testing process of the two scales is the same, the results are displayed together, but the analysis process was completely independent. The initial tests were based on the students of The China University of Geosciences (Beijing) as the survey sample.

The initial HRPS for college students had 5 dimensions and 15 items. From 4 November 2020 to 10 November 2020, a total of 170 questionnaires were collected in the initial test, and 129 valid questionnaires were screened, with an effective rate of 75.9%. The age range of the subjects was 18–28 years old (N = 129, M = 20.45, SD = 3.07), and gender was divided into male and female (N = 129, M = 1.46, SD = 0.512). Regarding the education level, 78.2% of the subjects were undergraduates, and 22.8% were masters and above. In order to simplify the expression in the analysis process, we used “a, b, c, d, e” to represent risk source perception, risk direct consequence perception, risk indirect consequence perception, risk liability perception and risk event perception, respectively, and numbers were used to distinguish each item.

The initial HRPIFS for college students had 4 dimensions and 15 items. From 7 November 2020 to 16 November 2020, a total of 260 questionnaires were collected in the initial test, and 210 valid questionnaires were screened, with an effective rate of 80.77%. The age range of the subjects was 18–30 years old (N = 210, M = 21.36, SD = 3.178), and gender was divided into male and female (N = 210, M = 1.31, SD = 0.501). Regarding the education level, 71.9% of the subjects were undergraduates, and 28.1% were masters and above. We used “A, B, C, D” to represent personal emotion, social trust, media communication and government policy, and numbers were used to distinguish each item.

#### 3.1.2. Item Analysis

In the initial HRPS, the 35 subjects at the top and bottom were regarded as the high group and the low group, respectively. The results of *t*-test showed that all items of the HRPS reached a significant level (*p* < 0.001, t > 3), which means that all items can effectively distinguish the subjects’ haze risk perception.

In the initial HRPIFS, the 57 subjects at the top and the bottom were regarded as the high group and the low group, respectively. The *t*-test showed that only the B1, B2 and B3 items had no significant difference between high and low groups (*p* > 0.05); these three items did not meet the standard of t > 3, as shown in Table 1. Therefore, the social trust dimension and the three items B1, B2 and B3 under this dimension were deleted.

#### 3.1.3. Exploratory Factor Analysis (EFA)

In EFA, the KMO values of the HRPS and HRPIFS were 0.714 and 0.818 (>0.6), respectively, and the chi-square statistics were also significant (*p* < 0.001), indicating that both datasets were suitable for factor analysis.

**Figure 3:**
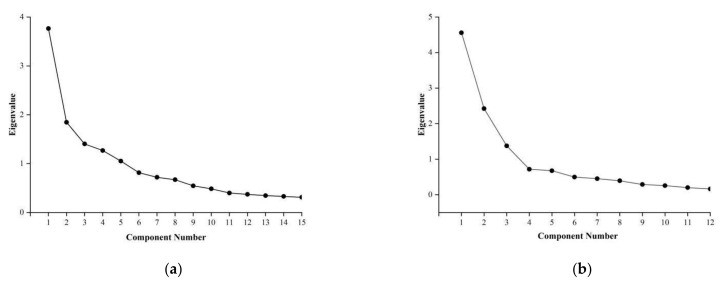
Scree plot of exploratory factor analysis. (**a**) Scree plot of HRPS; (**b**) Scree plot of HRPIFS.

We conducted factor analysis on 15 items of HRPS and judged the number of factors based on the explanatory variance rate and the scree plot. The results showed that five common factors could be extracted. The eigenvalues were 3.765, 1.847, 1.403, 1.268 and 1.053, respectively. The explanatory variance rates after rotation were 16.778%, 14.403%, 13.231%, 11.119% and 10.796%, respectively, accounting for 66.327% (>60%) of the total variation. Combined with the eigenvalue and scree plot (Figure 3a), the HRPS could be divided into five factors.

The results of factor analysis on ten items of HRPIFS showed that a total of three common factors could be extracted. The eigenvalues were 4.558, 2.423, and 1.375, respectively. The explanatory variance rate after rotation was 25.882%, 23.775% and 19.982%, and the cumulative percentage of the explanatory variance was 69.638% (>60%). Combined with the eigenvalue and scree plot (Figure 3b), the HRPIFS can be divided into three factors.

The varimax was used to obtain the factor loading matrix to assist in judging the number of factors. The results are shown in Table 2 and Table 3, respectively.

HRPS extracted a total of five principal components; d3 belonged to two dimensions, and the communality of d3 and d4 was not up to the standard (<0.5), so they were deleted. Both the factor loading and communality of the other items were greater than 0.5, which met the requirements of EFA. At this point, the HRPS contained five dimensions and 13 items.

As shown in Table 3, HRPIFS extracted a total of three principal components. The factor loading and communality of all items is more than 0.5. Therefore, after EFA, HRPIFS has three dimensions and 12 items.

#### 3.1.4. Reliability Analysis

From Table 4, it can be seen that the total reliability of the HRPS was 0.753, but the reliability value of the risk events dimension was 0.561 (<0.6). After deleting the risk event perception dimension as a whole, the reliability of the other dimensions of the questionnaire was improved, and they were all above 0.6. At this point, the HRPS had high internal consistency and strong reliability.

The reliability analysis results in Table 5 show that the overall reliability of the HRPIFS is 0.836, and the Cronbach’s Alpha value of each dimension is 0.771–0.889, which are all greater than 0.7. Therefore, we believe that the HRPIFS has high internal consistency and strong reliability.

After EFA and reliability analysis of HRPS, one dimension (risk event perception) was deleted, and five items (d2, d3, e1, e2, and e3) were deleted. The initial HRPS retains ten items and four dimensions. For HRPIFS, a total of one dimension (social trust) three items (B1, B2 and B3) were deleted. The initial HRPIFS retains 3 dimensions and 12 items.

### 3.2. Validation Results of the Scales

#### 3.2.1. Validation of HRPS and HRPIFS

Since the number of items of the adjusted scales had been significantly reduced, in order to improve research efficiency, we combined the HRPS and HRPIFS into one questionnaire in the validation survey. The validation results of the two scales are also displayed together, but the analysis process was completely independent. The questionnaires were distributed online, and the survey objects were college students in Beijing. The Beijing University Student Association Alliance helped us carry out this work.

From 23 November 2020 to 7 December 2020, a total of 461 questionnaires were collected, and 404 valid questionnaires were screened, with an effective rate of 87.64%. The age range of the subjects was 18–33 years old (N = 404, M = 22.71, SD = 3.204), and gender was divided into male and female (N = 404, M = 1.28, SD = 0.513). Regarding the education level, 65.3% of the subjects were undergraduates, and 34.3% were graduate students (masters and above).

There are 22 items in the questionnaire (10 items in HRPS and 12 items in HRPIFS), and as the 404 valid questionnaires are far more than ten times the number of items, the survey is suitable for CFA [46]. The HRPS still uses a, b, c and d to represent the risk source perception, direct consequences perception, indirect consequences perception and risk responsibility perception, respectively. The HRPIFS still uses A, C and D to represent personal emotion, media communication and government policy, respectively.

#### 3.2.2. Confirmatory Factor Analysis (CFA)

According to the factor load coefficient results in CFA, the standard estimated coefficient of the HRPS items was between 0.571 and 0.813 (>0.5), the CR values of the path coefficients were between 9.914 and 12.005, and the path coefficients were significant (*p* < 0.001). In the HRPIFS, the standard estimated coefficient of item A1, “I am very concerned about haze pollution,” was less than 0.5; this item needed to be deleted. The standard estimated coefficients of the remaining items were between 0.537 and 0.794 (>0.5), the CR value of the path coefficient was between 8.032 and 13.618, and the path coefficient was significant (*p* < 0.001). After deleting item A1, the CFA results in Table 6 show that the indicators of the two scales met the standards.

**Figure 4:**
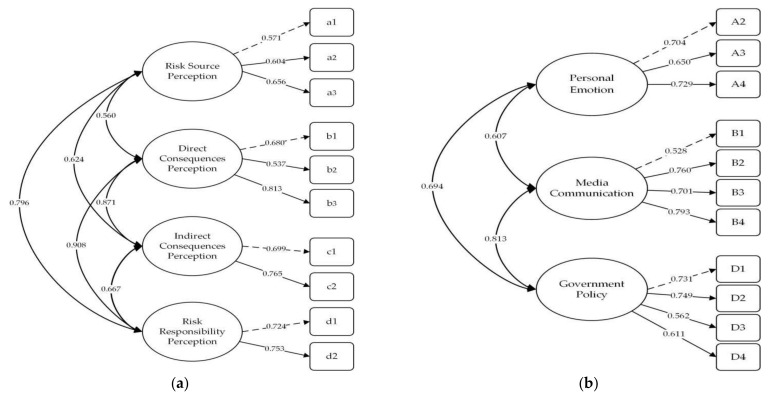
Standard path and parameter estimation model of CFA. (**a**) Model of HRPS; (**b**) Model of HRPIFS.

Through factor covariance analysis, the factors of both HRPS and HRPIFS were significant (*p* < 0.001), indicating that there was a specific correlation between the factors of the scale. Based on the new standard estimated coefficient and factor covariances, the model is shown in Figure 4.

#### 3.2.3. Reliability Analysis

The reliability analysis results of HRPS and HRPIFS are summarized in Table 7. The Cronbach’s Alpha of each dimension of HRPS and HRPIFS is above 0.6, and the overall reliability is more than 0.8, which indicates that the reliability of the two scales is excellent.

#### 3.2.4. Validity Analysis

There is often a common method variance (CMV) in surveys using questionnaires or scales, for which Harman’s single-factor test is usually used for testing [48]. The Harman’s single-factor test results of HRPS and HRPIFS showed that the number of factors with an eigenvalue greater than one extracted after rotation is four and three, and the most powerful factors can explain 16.778% and 25.882% of the total variation (<40%). The cumulative percentage of the explanatory variance of factors after rotation reached 66.327% and 69.638%, respectively (>50%), so there is no serious CMV problem in this study.

KMO and Bartlett’s test reflect the construct validity of the scale. The result is that the KMO values of HRPS and HRPIFS are 0.772 and 0.825 (>0.7), respectively, and Bartlett’s test is significant for both (*p* < 0.01), so the two scales have good construct validity. When preparing the questionnaire in the early stage, we fully referred to the relevant literature of the development scale and received suggestions from scholars in different industries. Therefore, the content validity of the scale meets the requirements. To prove this, participants did not show any doubts about the content of the questionnaire during the survey process. Table 8 shows the results of convergent validity. Table 9 and Table 10 show the results of discriminant validity.

The average variance extracted (AVE) of each dimension of the two scales is more than 0.5, and the combined reliability (CR) is more than 0.7. This shows that the two scales have good convergent validity.

The values in the above two tables represent the HTMT values between the two factors. All HTMT values are less than 0.85, which means that the factors have good discriminant validity.

#### 3.2.5. Determination of Formal Scales

After CFA, reliability analysis and validity analysis, all indicators of the two scales met the test requirements, and the scales were formally determined. The dimensions of HRPS were risk source perception, direct consequence perception, indirect consequence perception and risk responsibility perception. There were ten items in total. The three dimensions of HRPIFS were personal emotion, media communication and government policy. There were 11 items in total. The formal scales are in the Appendix A.

### 3.3. Results of Empirical Research

Taking college students in Beijing as the survey object, this paper did empirical research with the verified formal scale. The questionnaire was distributed online, and the Beijing University Student Association Alliance helped us carry out this work. From 27 January 2021 to 19 February 2021, a total of 1031 questionnaires were collected, and 706 valid questionnaires were screened, with an effective rate of 68.5%. The demographic information is shown in the Table 11.

#### 3.3.1. Analysis of the Status Quo of Haze Risk Perception

##### Perception of Haze Risk Source

Research on the source of PM_2.5_ in the air around Beijing shows that the primary source of haze is industrial emissions [3,49]. Our survey showed that in the five-level score of a3, “I know that the primary source of haze around Beijing is industrial emission,” 47.03% of the subjects thought “strongly agree” or “agree,” on the contrary, 32.82% thought “strongly disagree” or “disagree,”, and those who thought “unclear” accounted for 19.15%. This means that nearly half of the subjects did not know the fact that the main source of haze is industrial emissions, indicating that college students in Beijing do not have a high degree of haze risk source perception. However, for item a1, 67.31% of the subjects said they knew the causes of haze. This contradictory result shows that college students have a biased perception of the source of haze.

##### Perception of Direct Consequences of Haze

A report issued by the World Health Organization pointed out that haze not only affects the human respiratory system, but its main component, PM_2.5_, can have a toxic effect on multiple organs of the human body and can even induce serious diseases such as cancer [3,36]. When investigating the subjects’ perception of haze causing asthma, bronchitis and cardiovascular disease (b1), chronic disease (b2) and increasing mortality (b3), the sum of “very agree” and “agree” in the scores of each item was 90.49%, 71.43% and 75.99%, respectively, indicating that college students have a very high perception of the risk consequences of haze closely related to their health.

##### Perception of Indirect Consequences of Haze

In addition to direct harm to human health, haze can also affect the public’s daily activities, such as physical exercise [50]. Visibility is low in haze, which affects transportation [51]. The survey results of the indirect consequences of haze showed that 74.21% of college students believed that haze affects their outdoor activities, and 76.93% believed that haze hinders traffic. This shows that college students’ perception of the indirect consequences of haze is very high.

##### Perception of Haze Risk Responsibility

Clarifying the ownership and weight of responsibilities is key to risk governance [52]. This study evaluated the responsibility of government and enterprises for haze in China. In the questionnaire, 1–5 indicates the size of responsibility: the greater the score, the greater the responsibility. The empirical results show that the average score of government responsibility was 3.56, and the average score of enterprise responsibility was 4.04.

Industrial emissions around Beijing are the main source of haze [3]. Therefore, it is understandable that corporate responsibility ranks first, with a score of 4.04. At the same time, the Chinese government is the decision-maker and supervisor of relevant environmental protection policies, and it is responsible for environmental supervision. The government responsibility score was 3.56, which shows that the government has insufficient management of haze pollution and needs to bear certain responsibilities.

#### 3.3.2. Analysis of Influencing Factors of Haze Risk Perception

The three dimensions of HRPIFS were used as independent variables, and the overall risk perception was used as the dependent variable. The results of bivariate correlation analysis are shown in Table 12. It can be seen that there is a very significant positive correlation between personal emotion, media communication and total risk perception (*p* < 0.001), and there is a significant positive correlation between government policy and total risk perception (*p* < 0.05). This conclusion is consistent with the previous theoretical model, indicating that personal emotion, media communication and government policy all affect the haze risk perception of college students.

In order to study whether personal emotion, media communication and government policy have an effect on various dimensions of HRPS, one-way ANOVA was used to compare the differences in the mean of each group.

##### Personal Emotion

We took each dimension of the HRPS as the dependent variable, and the score of A1, “I am always afraid that haze will harm my health”, of the personal emotion dimension as the grouping variable. ANOVA was used to compare the differences of each personal emotion grouping and each dimension of risk perception. The results are shown in Table 13 below.

**Figure 5:**
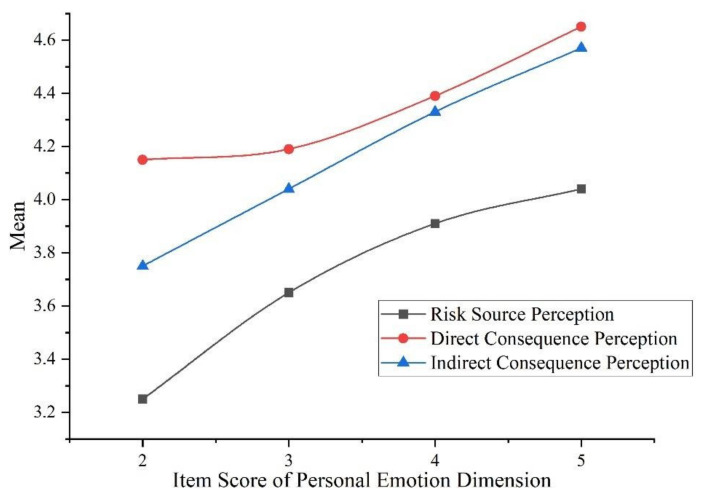
The relationship between personal emotion and risk perception.

It can be seen from the mean values in Table 13 that, at the same level of concern, college students have the highest perception of direct consequences, followed by indirect consequence perception, risk responsibility perception and risk source perception. The results of ANOVA showed that personal emotion creates significant differences in risk source perception, direct consequence perception and indirect consequence perception (*p* < 0.05). We compared the mean values in the dimensions with significant differences; the relationship is shown in Figure 5.

The above figure intuitively shows a positive correlation between personal emotion and haze risk perception among college students. In terms of this item, the more concerned college students are about haze, the higher their perception of risk source, direct and indirect consequences.

##### Media Communication

**Figure 6:**
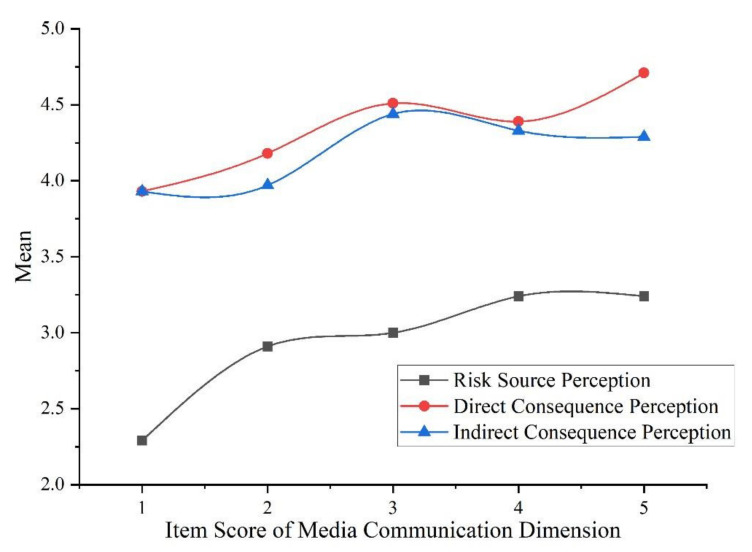
The relationship between media communication and risk perception.

Using the same method as in the previous section, item C1, “I think the media has played an important role in popularizing the scientific knowledge of haze”, of the dimension of media communication was taken as the grouping variable. The results are, that among the five levels of media communication effects, the degree of risk perception ranked from high to low is: direct consequence perception, indirect consequence perception, risk responsibility perception and risk source perception. The results of ANOVA showed that media communication has significant differences in risk source perception, direct consequence perception and indirect consequence perception (*p* < 0.05). We compared the mean values in the dimensions with significant differences; the relationship is shown in Figure 6.

The above figure shows a positive correlation between media communication and college students’ haze risk perception. In terms of this item, with the enhancement of the media communication effect on haze-related knowledge, college students’ perception of risk source, direct consequences and indirect consequences increases.

##### Government Policy

**Figure 7:**
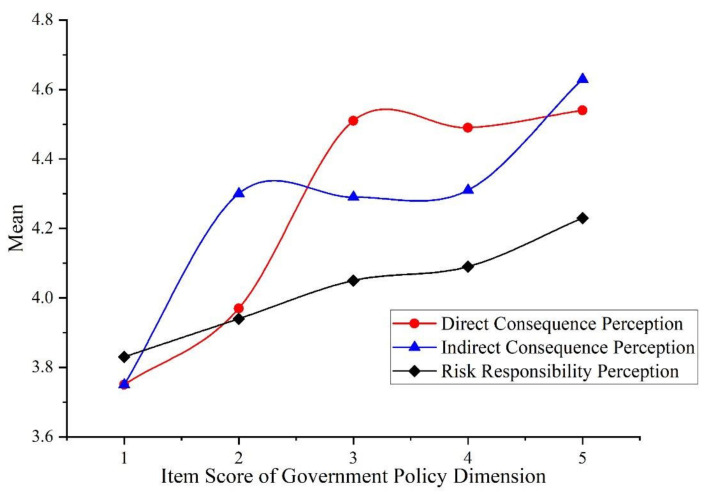
The relationship between government policy and risk perception.

Taking item D1, “Strict government supervision of the behavior of heavily polluting companies can improve air quality”, of the dimension of government policy as the grouping variable, the results are that in the dimension of government policy, the ranking of haze risk perception is the same as the other two influencing factors. The perception degree from high to low is: direct consequence perception, indirect consequence perception, risk responsibility perception and risk source perception. The results of ANOVA showed that government policy has significant differences in the dimensions of direct consequence perception, indirect consequence perception and risk responsibility perception (*p* < 0.05). We compared the mean values in the dimensions with significant differences; the relationship is shown in Figure 7.

The figure shows a positive correlation between government policy and college students’ haze risk perception. In terms of this item, with the enhancement of the implementation of haze control policies, college students’ perception of the direct consequences, indirect consequences and risk responsibilities increases.

## 4. Discussion

### 4.1. Summary of Scales

This study explores the reliable structure of haze risk perception and its influencing factors of college students in Beijing. The findings enrich theories related to risk perception and its influencing factors. Compared to previous scale studies that developed a single construct, our research process and results are more systematic and explanatory, with certain theoretical contributions. From a practical point of view, our research can guide college students to form rational risk perception and can also provide a decision-making reference for improving the collaborative social risk governance system.

Classic risk perception has been mostly measured from the dimensions of dread risk and unknown risk [17]. The influencing factors of risk perception summarized in previous studies include risk preference, government support and social justice [21], or the level of social development, information communication channels and the degree of haze hazard [53]. The HRPS and HRPIFS formed in this paper are different from previous scales in structure and content. The reasons for this difference are multifaceted. First, our research was conducted in a group of college students, whose natural and spiritual attributes may affect the dimension of psychological cognition. Second, national and regional differences cannot be ignored. As a special environmental risk, haze mainly appears in developed regions of developing countries. Compared to developed and underdeveloped countries, the social attributes of college students in Beijing are unique. It is precisely because college students in the Beijing area are characterized by high education and rich haze experience that their attitude towards haze can reflect the current social situation. This is one of the reasons why college students in Beijing were chosen as the research object in this study.

From the reliability analysis results, the reliability of the HRPS and the HRPIFS are 0.835 and 0.819, respectively, indicating that the scales have strong internal consistency. The effective response rates of both scales exceeded 68.5% in all four surveys. Therefore, judging from the response to the scales, the content validity of the two scales met the requirements. In the validation stage, we proved that the two scales had good construct validity, convergent validity and discriminant validity. In order to reduce the CMV, our subjects were multi-source, and the survey was conducted at different stages and periods. The CMV test results show that the factors with the greatest explanatory power in HRPS and HRPIFS could explain 16.778% and 25.882% (<40%) of the total variation, respectively. The cumulative percentage of the explanatory variance of factors after rotation reached 66.327% and 69.638% (>50%), respectively, so there is no serious CMV problem in this study. The above summary suggests that HRPS and HRPIFS can be used as effective tools to measure the public’s, especially college students’, attitude towards haze.

### 4.2. Discussion of Empirical Research Results

With the rapid development of big data technology and the internet, the assessment methods for haze risk perception become more diverse. For example, risk perception can be indirectly represented by network heat and haze concentration data [27,53]. However, official data based on objective indicators such as pollutant concentrations are not widely accepted by individuals or the public [54]. From the perspective of college students, their perceptions towards haze risk are still affected by their personal experiences and social environment. Therefore, it is still of positive significance to reflect the risk perception and related influencing factors of college students in Beijing through the measurement scales.

The empirical research found that personal emotion, media communication and government policy were significantly and positively correlated with total risk perception, which is consistent with our constructed theory. In terms of the average value of project scores, the degree of risk perception ranked from highest to lowest is: direct consequence perception, indirect consequence perception, risk responsibility perception and risk source perception. We believe that this result is consistent with the process by which people understand risk. When facing haze risk, the first feeling or information that college students get is the direct damage of the haze to themselves, followed by indirect risk consequences. Due to loss or injury, they will think further about who should be responsible for this risk and finally explore the source of the risk to eliminate it. The haze perception reaction chain proposed by Jiuchang Wei is similar to the above results. He and his co-workers believe that the haze experience leads to consequence perception, consequence perception leads to attribution of responsibility, and attribution of responsibility leads to intention to take protective actions [55].

Figure 5, Figure 6 and Figure 7 obtained from the variance analysis show the relationship between personal emotion, media communication and government policy to the dimensions of haze risk perception. Figure 5 shows that the personal emotion of college students can significantly affect risk source perception, direct consequences perception and indirect consequences perception. Specifically, the more concerned college students are about haze, the higher their risk perceptions are. The more they care about the value of PM_2.5_, the higher their risk perceptions. This is well-understood. From a psychological point of view, emotion is a key factor affecting attitude [56]. Research using Weibo as the source of emotional data showed that negative emotions significantly enhanced public risk perceptions in both the COVID-19 pandemic and haze [22,57].

Figure 6 showed that enhancement of the media’s effect on haze knowledge dissemination increased college students’ haze risk perception. Regarding the role of the media in disaster situations, scholars hold two views. One view is that the media has a positive significance for the public to scientifically recognize risks, while the other view is that the media can intentionally or unintentionally cause risk information to be distorted in the process of dissemination, thereby misleading public opinion and public risk perception [58,59]. These two views may seem to be contradictory, but they do not conflict with the results of this research. When media communication plays a positive role, it can promote public perception of risk source and risk responsibilities. When media communication plays a negative role, it tends to amplify the risk consequences and cause public panic. According to the theory of this study, these two scenarios will increase public risk perception.

Figure 7 shows that the implementation of haze control policies increased college students’ haze risk perception. At present, Beijing has adopted many policies and measures for haze control, such as eliminating and relocating some high-energy-consuming and high-polluting enterprises around Beijing, and encouraging citizens to travel green [30]. From our survey results, these policies have indeed improved the public’s perception of haze risk, but the significance level and correlation coefficient of government policy and risk perception are significantly lower than the other two dimensions. We believe that this is related to the short-term and long-term effects of the policy [60]. When a new environmental protection policy emerges, it may attract a lot of social attention in the short term, which is reflected in the significant increase of risk perception. However, over time, the influence of the policy declines, and with implementation of the policy, air quality improves, so the public’s risk perception will weaken or be reduced [30].

### 4.3. Enlightenment of Haze Governance

For a developing country such as China, prevention and control of haze is a protracted battle. We have put forward some policy suggestions by exploring haze risk perception and influencing factors of college students in Beijing.

Nowadays, it is increasingly common for the public to obtain risk information through online channels such as Facebook, Twitter and Weibo, and to generate a lot of public opinions [22]. Public opinion is a double-edged sword. On the one hand, public opinion can pressure the government to improve air quality in the region in the short term [26]. On the other hand, over-reliance on online media can lead to distortion of information, especially risk information [58]. For example, in order to attract attention and get more clicks, some media will wantonly exaggerate the severity of haze. To a certain extent, this has a misleading effect on the haze risk perception among college students. The results of this study showed that 52.97% of the subjects did not know that the primary source of haze around Beijing was industrial emissions, although they were very concerned about the direct or indirect consequences of haze. Well-educated college students still have a misunderstanding about haze, and the public with a lower level of education, or the elderly and children, may have a lower haze risk perception. Therefore, the media, especially the official media, should actively take the responsibility of communicating with the public and guide the public to form a scientific and rational risk perception through extensive popularization of science [61].

In China, the government—with absolute authority—is an important participant and leader in haze governance. Therefore, the implementation of policies related to haze will have an important impact on the effectiveness of haze governance [30]. In order to ensure the government’s credibility and the scientific nature of the decision making, we suggest that the government do the following: First, in the process of haze governance, the government must severely crack down on enterprises that do not meet air emissions standards. The process and results of haze governance should be announced on the official website in real-time. Second, the government must fully consider public perception of haze risks and influencing factors. Relevant policies and measures should not only meet the needs of haze governance, but also adapt to the status quo of public perception of haze risks. Third, the government should improve early-warning and late-release mechanisms of air quality. In particular, the government should do a good job in tracking and controlling risk information related to haze on the internet, timely curb the spread of false information on the internet, and reduce public panic.

### 4.4. Limitations and Future Research Directions

This study not only compiled the HRPS and HRPIFS of college students in Beijing through three administrations of a questionnaire survey, but also measured the realistic level and impact mechanism of haze risk perception in an empirical study. However, this study also has limitations. First of all, this study effectively reduced the common method bias through multi-period surveys, but the online survey method makes it difficult to ensure the representativeness of the subjects. Second, the severity of haze varies with seasons, and this study did not consider the impact of seasonal factors on college students’ haze risk perception. We suggest that future research combines objective data and psychometrics to comprehensively analyze the risk perception status of college students through real-time PM_2.5_ concentration and social media interaction data. In addition, follow-up research can reveal the changing laws of risk perception and related influencing factors through survey data with a longer time span. Finally, using the scales developed in this study to evaluate risk perception and influencing factors of college students in different regions may result in more interesting and meaningful findings.

## 5. Conclusions

This study developed and verified HRPS and HRPIFS in strict accordance with the scale development steps, and both scales were proven to be reliable and valid. The HRPS for college students has ten items, which can be divided into four dimensions, ranked from perception of high to low risk as: risk source perception, direct consequence perception, indirect consequence perception and risk responsibility perception. The HRPIFS for college students has eleven items, which can be divided into three dimensions, ranked from high to low according to the strength of their influence as: personal emotion, media communication and government policy. Although college students are very concerned about the direct and indirect consequences of haze, their perception of haze risk source is not high and there is perception bias. College students believe that enterprises bear the primary responsibility for haze and the government bears an important responsibility. Personal emotion, media communication and government policy will have a significant positive impact on the overall haze risk perception. Among them, personal emotion and media communication are significantly positively correlated with the three dimensions of risk source perception, direct consequence perception and indirect consequence perception, while government policy is significantly positively correlated with the dimensions of direct consequence perception, indirect consequence perception and risk responsibility perception. The two scales can accurately measure the dimension, degree and internal differences of haze risk perception and influencing factors of college students in Beijing. The research results are helpful for policy makers to guide college students to form rational risk perception, and can also provide a decision-making reference for improving social risk governance, which has important theoretical and practical significance.

## Figures and Tables

**Table 1:** Extreme Group *t*-test Results for Identification of Unqualified Items.

		Levene Test		*t*-Test						
		F	Sig	T	*df*	*p*	Mean Difference	Std. Error	95% Confidence Interval for Difference	
									Lower	Upper
B1	Equal Variance	0.110	0.741	2.348	129	0.020	0.537	0.229	0.990	0.084
	Unequal Variance			2.369	128.493	0.019	0.537	0.227	0.986	0.088
B2	Equal Variance	2.394	0.124	1.582	129	0.116	0.292	0.185	0.658	0.073
	Unequal Variance			1.565	118.625	0.120	0.292	0.187	0.662	0.078
B3	Equal Variance	0.768	0.383	1.811	129	0.073	0.331	0.183	0.694	0.031
	Unequal Variance			1.809	125.002	0.073	0.331	0.183	0.694	0.031

**Table 2:** Rotated factor loading matrix (HRPS).

Item	Factor 1	Factor 2	Factor 3	Factor 4	Factor 5	Communality
d1	0.868					0.824
d2	0.826					0.803
d3	0.415	0.519				0.431
d4	0.521					0.469
c1		0.827				0.810
c2		0.791				0.767
b1			0.780			0.792
b2			0.727			0.657
b3			0.680			0.618
a1				0.789		0.769
a2				0.689		0.617
a3				0.688		0.710
e1					0.810	0.706
e2					0.641	0.621
e3					0.575	0.599

**Table 3:** Rotated factor loading matrix (HRPIFS).

Item	Factor 1	Factor 2	Factor 3	Communality
A1	0.823			0.769
A2	0.798			0.732
A3	0.738			0.720
A4	0.702			0.663
C1		0.847		0.809
C2		0.844		0.786
C3		0.834		0.817
C4		0.699		0.690
D1			0.885	0.852
D2			0.865	0.781
D3			0.863	0.708
D4			0.758	0.622

**Table 4:** Reliability Analysis Results of the HRPS.

	Risk Event Perception	Risk Source Perception	Direct Consequences Perception	Indirect Consequences Perception	Risk Responsibility Perception	HRPS
Cronbach’s Alpha	0.561	0.723	0.697	0.624	0.630	0.753
Number of Items	3	3	3	2	2	13

**Table 5:** Reliability Analysis Results of the HRPIFS.

	Personal Emotion	Media Communication	Government Policy	HRPIFS
Cronbach’s Alpha	0.771	0.867	0.889	0.836
Number of Items	4	4	4	12

**Table 6:** The fitted result of the CFA.

Fitting Index	X^2^/*df*	GFI	NFI	CFI	TLI	RMSEA
Fitted Value (HRPS)	3.530	0.961	0.909	0.940	0.932	0.037
Fitted Value (HRPIFS)	3.957	0.918	0.932	0.944	0.917	0.031
Standard Value	<5	>0.9	>0.9	>0.9	>0.9	<0.1

**Table 7:** Results of reliability analysis.

	Cronbach’s Alpha	Number of Items	Scale
Risk Source Perception	0.779	3	0.835
Direct Consequences Perception	0.761	3	
Indirect Consequences Perception	0.712	2	
Risk Responsibility Perception	0.680	2	
Personal Emotion	0.755	3	0.819
Media Communication	0.861	4	
Government Policy	0.824	4	

**Table 8:** Results of convergent validity.

Factors	AVE	CR
Risk Source Perception	0.679	0.760
Direct Consequences Perception	0.631	0.825
Indirect Consequences Perception	0.618	0.804
Risk Responsibility Perception	0.593	0.736
Personal Emotion	0.586	0.781
Media Communication	0.647	0.799
Government Policy	0.692	0.834

**Table 9:** The result of HTMT about HRPS.

	Risk Source Perception	Direct Consequence Perception	Indirect Consequence Perception	Risk Responsibility Perception
Risk Source Perception	-			
Direct Consequence Perception	0.461	-		
Indirect Consequence Perception	0.569	0.608	-	
Risk Responsibility Perception	0.510	0.522	0.594	-

**Table 10:** The result of HTMT about HRPIFS.

	Personal Emotion	Media Communication	Government Policy
Personal Emotion	-		
Media Communication	0.577	-	
Government Policy	0.490	0.423	-

**Table 11:** Demographic Information of Empirical Research.

Variables	Classification	Number	Percentage (%)
Gender	male	394	55.81
	female	312	44.19
Age	≤20	69	9.77
	21–25	398	56.37
	26–30	199	28.19
	≥31	40	5.67
Education Level	Junior college degree	27	3.82
	Bachelors degree	417	59.07
	Masters degree	212	30.03
	PhD degree	50	7.08

**Table 12:** Bivariate Correlation Analysis.

		Personal Emotion	Media Communication	Government Policy
Haze Risk Perception	Pearson	0.343 **	0.278 **	0.176 *
	Sig.	0.000	0.000	0.028
	N	706	706	706

Note: ** indicate *p* < 0.01, * indicate *p* < 0.05.

**Table 13:** Differences of haze risk perception in the dimension of personal emotion.

Personal Emotion	Risk Source Perception	Direct Consequence Perception	Indirect Consequence Perception	Risk Responsibility Perception
1	0	0	0	0
2	3.24	4.15	3.75	4.00
3	3.65	4.19	4.04	3.74
4	3.91	4.39	4.33	4.07
5	4.04	4.65	4.57	4.22
Total	3.88	4.42	4.32	4.05
ANOVA	F = 4.537, *df* = 706, *p* = 0.004	F = 3.965, *df* = 706, *p* = 0.008	F = 3.404, *df* = 706, *p* = 0.018	F = 2.250, *df* = 706, *p* = 0.082

Note: 1–5 respectively indicate strongly disagree to strongly agree, and in this usage a higher number means the degree of concern is increasing.

## Data Availability

The data presented in this study are available on request from the corresponding author. The data are not publicly available due to privacy or ethical concerns.

## Appendix A

**Table A1:** Formal HRPS and HRPIFS for college students.

Haze Risk Perception Scale (HRPS)	
Risk Source Perception	I know the source of haze
	Haze is the result of the combined influence of meteorological and man-made causes, among which man-made environmental pollution is the main reason
	The primary source of haze around Beijing is industrial emission
Direct Consequence Perception	Haze can cause diseases such as asthma, bronchitis and cardiovascular disease
	Haze can cause chronic diseases
	Haze will increase mortality
Indirect Consequence Perception	Haze will affect my outdoor activities
	Haze will hinder air, water and road traffic
Risk Responsibility Perception	I think the government should take important responsibility for the haze weather
	I think enterprises take important responsibility for the haze weather
**Haze risk perception influencing factors scale (HRPIFS)**	
Personal Emotion	I am always afraid that haze will harm my health
	I am very concerned about the value of PM_2.5_ in the air
	I feel depressed in haze weather
Media Communication	I think the media has played an important role in popularizing the scientific knowledge of haze
	I think the media is very effective in promoting the formulation and implementation of haze governance policies
	I think the media is very effective in improving the public’s haze risk perception
	I think the media is very effective in advocating a low-carbon life for the public
Government Policy	The government’s strict supervision of heavily polluting enterprises can improve air quality
	The government imposes production restrictions and cessation of production on enterprises that violate regulations, which can improve air quality
	Central government supervision of local governments can improve regional air quality
	The government has played an important role in eliminating high energy-consuming and high-polluting enterprises around Beijing
